# Impact of the use of food ingredients and additives on the estimation of ultra-processed foods and beverages

**DOI:** 10.3389/fnut.2022.1046463

**Published:** 2023-01-10

**Authors:** Camila Zancheta Ricardo, Ana Clara Duran, Mariana Fagundes Grilo, Natalia Rebolledo, Ximena Díaz-Torrente, Marcela Reyes, Camila Corvalán

**Affiliations:** ^1^Doctoral Program in Public Health, School of Public Health, University of Chile, Santiago, Chile; ^2^Center for Food Studies and Research, University of Campinas, Campinas, Brazil; ^3^Center for Epidemiological Studies in Nutrition and Health, University of São Paulo, São Paulo, Brazil; ^4^Institute of Nutrition and Food Technology, University of Chile, Santiago, Chile; ^5^Doctoral Program in Nutrition and Food, University of Chile, Santiago, Chile

**Keywords:** children, NOVA classification, food additives, ultra-processed food, food processing, child diet, Chile

## Abstract

**Introduction:**

Increasing consumption of ultra-processed foods (UPF), defined by the NOVA classification, has been associated with obesity and other health outcomes. However, some authors have criticized the UPF definition because it is somewhat subjective. Most studies identify UPF using food descriptions; nevertheless, NOVA developers described a list of ingredients, including substances not commonly used for cooking and “cosmetic additives” that could be used to identify UPF. Assessing the impact of the use of different UPF definitions is particularly relevant with respect to children’s diet, because several dietary policies target this age group. Thus, our study compared the frequency of UPF among foods and beverages and their share in the diet of Chilean preschoolers using three different methods of identifying UPF.

**Methods:**

We used cross-sectional 24-h dietary recall data from 962 preschoolers enrolled in the Food and Environment Chilean Cohort (FECHIC) in 2016. All foods and beverages consumed were classified according to NOVA, considering their description (classic method), the presence of ingredients markers of UPF (ingredient marker method), and the presence of markers plus all cosmetic additives (food additive method). We also estimated the caloric share and quintiles of UPF consumption using the three methods. We used kappa coefficients, consistency-of-agreement intra-class correlation (CA-ICC), absolute agreement intra-class correlation (AA-ICC), and weighted kappa coefficients for assessing agreement between methods.

**Results:**

The proportion of UPF products were 65% in the “classic,” 67% in the “ingredient marker,” and 73% in the “food additive” method, and kappa coefficients between methods varied from 0.79 to 0. 91. The caloric share of UPF was 47, 52, and 58% with “classic,” “ingredient marker,” and “food additive” methods, respectively. Consistency-of-agreement was higher than the absolute agreement between the methods (CA-ICC = 0.81; AA-ICC = 0.74). For quintiles of UPF consumption, we found weighted kappa of 0.65 as measure of agreement between “classic” and “ingredient marker,” and 0.51 between “classic” and “food additive” methods.

**Conclusion:**

Searching for all possible markers of UPF in the list of ingredients increased the proportion of food products identified as UPF compared to the classic method. These differences affected the estimated caloric share of UPF in Chilean preschoolers’ diets.

## 1. Introduction

The growing prevalence of obesity and non-communicable diseases worldwide is associated with changes in the food system, the weakening of traditional eating patterns, and the increasing participation of packaged and ready-to-eat products in the diet (1). Several classification systems considering food processing have been proposed, with the NOVA food classification system being the most extensively used (2). NOVA classifies all food and beverages into four groups: minimally processed foods (MPF), processed culinary ingredients (PCI), processed foods (PF), and ultra-processed foods (UPF) (3).

Nutritional epidemiologists are increasingly using the NOVA classification system (1), which has already been applied to food purchase data (4), in national food consumption surveys (5–8), cohort studies (9–11), and a randomized controlled trial (12). Furthermore, several systematic reviews show that higher UPF consumption is associated with obesity and non-communicable diseases, especially in adults (13–15). The concept of UPF also appears in the nutrition profile model proposed by the Pan American Health Organization (16) and various dietary guidelines (17–20), which recommend diminishing or avoiding the consumption of these products.

NOVA aims to classify foods and beverages considering the extent and purpose of industrial processing, and its first versions were mainly based on the description of food categories that compose each group (21, 22). According to this definition UPF are generally ready-to-eat industrial formulations made from various food-derived ingredients, many exclusively used by the food industry, combined with food additives through various industrial processes. However, some foods such as breads or cheeses can be considered processed or UPF so NOVA classification has been criticized because the UPF definition is considered somewhat arbitrary (23, 24). For those cases, NOVA developers proposed a method to identify a UPF using the list of ingredients. They stated that it is possible to identify a UPF by the presence of food substances never or rarely used in traditional recipes or by the presence of functional classes of additives used to make a product palatable or more appealing—which they defined as “cosmetic additives” (3).

The extensive use of ingredients to support the application of NOVA should result in a more objective classification, potentially reducing misclassification. Assessing the impact of the use of different UPF definitions is particularly relevant with respect to children’s diet, because they are high UPF consumers (5, 8, 25, 26), and several dietary policies are targeted to this age group (27, 28). Thus, in the current study, we took advantage of detailed data on food composition and dietary intake from a cohort of Chilean preschoolers to compare the use of three different methods to identify UPF (“classic,” “ingredient marker,” and “food additives”). To our knowledge, no published work has made such comparisons.

## 2. Materials and methods

### 2.1. Design and subjects

We conducted a secondary cross-sectional analysis using baseline data from the Food Environment Chilean Cohort (FECHIC), a cohort study initiated in 2016 with 962 3-6-year old low-to-middle income preschoolers from Southeast Santiago, Chile, to assess changes in dietary intake after the Chilean Law of Food Labeling and Advertising (27). We included all children with dietary data available for 2016 (*n* = 958) for the current analyses. Details on recruitment and inclusion criteria are available elsewhere (29).

### 2.2. Ethics

The ethics committees of the Institute of Nutrition and Food Technology (INTA) and the School of Public Health, University of Chile, approved this study. Mothers signed the informed consent on behalf of their children.

### 2.3. Dietary intake

Trained dietitians collected 24-h dietary recalls following the United States Department of Agriculture (USDA) Automated Multiple-Pass method (30). They entered data on portion size, type of preparation, type of food, and product brand and flavor in the case of packaged foods, besides the source of the food and eating location, directly in SER-24, a software developed by the Center for Research in Food Environments and Prevention of Nutrition-Associated Diseases (CIAPEC), INTA (29, 31). A photographic food atlas was used to estimate the portion size consumed accurately (29). The primary caretaker was responsible for reporting the diet, and the children complemented the information for the occasions when the respondent was absent (e.g., school time).

### 2.4. NOVA food classification system

Briefly, NOVA considers the extent and purpose of industrial processing and classifies each food and beverage into one of four exclusive groups: Group 1. Minimally processed foods (MPF) are defined as whole foods or parts of foods not modified or that have undergone only processes aimed at facilitating preservation, storage, or consumption. In general, there is no inclusion of new ingredients. MPF includes grains, vegetables, milk, meats, eggs, seeds, and nuts; Group 2. Processed culinary ingredients (PCI) are substances extracted or collected in nature and primarily used in food preparation, such as salt, sugar, butter, oils, and vinegar; Group 3. Processed foods (PF) are combinations of minimally processed foods with culinary ingredients. PF includes salted meats, fish and canned vegetables, fruit compotes, and artisanal types of bread and cheese; Group 4. Ultra-processed foods (UPF) are generally ready-to-eat industrial formulations made from various food-derived ingredients, many exclusively used by the food industry, combined with food additives through various industrial processes. Examples of UPF are industrialized sodas, confectionaries, chocolates, ice cream, hamburgers, sausages, and other reconstituted meat products, pizzas and other frozen dishes, instant soups, cookies, cakes, and different types of packaged bread, among others (3).

We used different methods to identify UFP based on the NOVA food classification system.

#### 2.4.1. Classic method to identify UPF using NOVA classification system

We identified all unique foods and beverages consumed by children reported in the 24-h dietary recalls (*n* = 1,861) and categorized each of them according to the degree of processing in one of the four mutually exclusive groups defined by the NOVA classification. The developers of NOVA have previously described this NOVA classification method for large data sets (32–34). In the classic method, each food and beverage was classified considering the following information: group and type of food, packaged or unpackaged, and brand and flavor, when available. Simple preparations included in the software SER-24 (e.g., cooked rice or fried egg) were classified based on their main component. We disaggregated more elaborate homemade recipes into their components, and each of them was individually classified. Unbranded traditional Chilean bread was classified as PF and industrially produced, packaged, branded bread as UPF. Ready-to-eat meals such as pizza, hamburgers, and hotdogs purchased in supermarkets or fast-food chains were considered UPF.

The food classification process was carried out by a postgraduate dietitian at CIAPEC and reviewed by a second dietitian. Disagreements (0.4%) were discussed and resolved by consensus. A third rater classified a random subset of 5% of SER-24 records to verify the agreement between evaluators. We found an agreement of 97.4% and a kappa coefficient of 0.95, indicating almost perfect agreement between raters for the classic method of NOVA classification.

#### 2.4.2. Ingredient marker method to identify UPF using NOVA classification system

In this method, the lists of ingredients of 1,449 packaged foods (98.8% of all consumed packaged foods) were used by linking SER-24 records with information from food labels collected in supermarkets in Santiago in 2015 and 2016 [details of data collection are published elsewhere (35)]. The database linking procedure was performed manually by trained research assistants using the descriptive information available in the dietary data (36). We considered a product as UPF when it declared at least one ingredient not commonly used in home cooking or at least one class of additive that could modify its sensorial characteristics (or a “cosmetic additive”), according to NOVA developers (3). Monteiro et al. (3) presented a list of ingredients that would be exclusively used in UPF, including different sources of sugars, carbohydrates, proteins, and fats (i.e., non-additive markers of UPF); classes of food additives; and some specific names of these additives that consumers could commonly recognize. Based on this recommendation and the examples displayed in their publication, we searched the lists of ingredients for the following terms (in Spanish): inverted sugar, dextrose, fructose, lactose, glucose, maltodextrin, concentrated juice, syrup, protein concentrated, protein isolate, whey protein, soy protein, wheat gluten, casein, fiber, maltitol, sorbitol, interesterified, hydrogenated or fractionated oil/fat, gelatin, pectin, gums, mechanically separated meat, milk whey, dairy product solids, modified starch, monosodium glutamate, artificial essence/flavor, sucralose, aspartame, acesulfame, cyclamate, saccharin, Stevia, flavoring, flavor enhancer, color, emulsifier, emulsifying salt, sweetener, thickener, and antifoaming, bulking, carbonating, foaming, gelling and glazing agents. Packaged products that did not include these ingredients were reviewed and classified into the remaining NOVA food groups based on their description (our classic method) (*n* = 322).

We did not have access to the list of 412 foods and beverages (22.1% of total products), which remained in the NOVA group defined by the classic method. Most of them were minimally processed foods that do not have a list of ingredients such as fruit, vegetables, meats, eggs, grains, water, and herbs for tea (63.4%). We also did not obtain information on ingredients for unpackaged bakery products (13.8%), some processed meat and cheeses (7.8%), prepared foods and desserts from fast food chains (5.6%), and products provided by the Chilean Ministry of Health or the school meal program (3.4%).

#### 2.4.3. Food additive method to classify foods and beverages using NOVA classification system

The third method to identify UPF was also applied to the packaged products that had a list of ingredients (*n* = 1,449). Besides all ingredient markers of UPF previously described, we included additives’ specific names in this method. Using the list of ingredients of each product, we searched for all 388 additives defined by Codex Alimentarius (37). In addition to the standardized names, we included in the search terms synonyms described in the Chilean Food Sanitary Regulation (38) and other synonyms, mistyping, and codes of the International Numbering System (INS) found in the dataset. We considered a food additive as cosmetic if it could assume any of the 12 functional classes described by Monteiro et al. (3): flavor enhancer, color, emulsifier, emulsifying salt, sweetener, thickener, and antifoaming, bulking, carbonating, foaming, gelling and glazing agents; or if it was a flavoring (not specified as a functional class in Codex). For example, in this method we searched the lists of ingredients for the term that describes a functional class (e.g., emulsifier) and also for all 122 specific additives that can assume this function (e.g., lecithin, acetic and fatty acid esters of glycerol, agar, carrageenan, propylene glycol, among others). We applied the same method for other classes of cosmetic additives. Products that did not contain those additives remained in the NOVA group previously defined by the “ingredient marker method.”

### 2.5. Food composition table and food categories

We used an updated food composition table created with the nutrition facts panel for the packaged foods consumed by the children as described elsewhere (36). For unpackaged foods, we maintained the nutritional information available at the SER-24 (39).

Each food and beverage were categorized following the approach of previous studies that applied NOVA (26, 33, 40), considering some required adjustments. Final food categories were: (1) water, tea, and coffee; (2) soft drinks; (3) milk and plain yogurt; (4) milk-based drinks; (5) flavored or sweetened yogurt; (6) dairy desserts; (7) cheese; (8) cereals, flours, and pulses; (9) breakfast cereals and granola bars; (10) fresh breads; (11) packaged breads; (12) crackers and cookies; (13) cakes and pies; (14) snacks; (15) confectionaries; (16) fast food; (17) soups, sauces, and salts; (18) meat, fish and eggs; (19) salted, smoked or canned meat or fish; (20) reconstituted meat or fish; (21) fruits and vegetables; (22) fruits and vegetable preserves; (23) baby food; (24) sweeteners; (25) fats and oils. Supplementary Table 1 presents the description of each food category.

### 2.6. Statistical analysis

For food and beverages identified on dietary recalls, we calculated the proportion of NOVA groups by dividing the number of foods and beverages in each group by the total number of unique products in the database. We used kappa coefficients to assess the agreement between the different methods of NOVA classification. We considered the following thresholds for kappa values: less than 0.20, between 0.21 and 0.40, between 0.41 and 0.60, between 0.61 and 0.80, and above 0.81 as slight, fair, moderate, substantial, and almost perfect, respectively (41). We also described differences in the proportion of UPF identified using each method by food categories.

As a sensitivity analysis, we verified the agreement between methods in food and beverages only considering the packaged products with a list of ingredients (*n* = 1,449) since the unpackaged products were kept in the same NOVA food groups defined using the food description.

For children’s consumption, we estimated the caloric share of UPF (UPF kcal/total kcal) in the diet for each participant and the overall mean caloric share using each classification method. We also predicted the probability density of caloric share of UPF for each method with kernel density estimation. Additionally, we ranked UPF consumption into quintiles, with the lowest consumers in the first quintile and the highest consumers in the fifth. Agreement between the caloric share of UPF obtained by the three methods was estimated using a two-way mixed effects model, estimating absolute-agreement intra-class correlation (AA-ICC) and consistency-of-agreement (CA-ICC) (42). We considered the following thresholds for ICC values: less than 0.5, between 0.51 and 0.75, between 0.76 and 0.90, and greater than 0.91 as poor, moderate, good, and excellent agreement, respectively (42). We used linear weighted kappa to assess the agreement between quintiles of UPF consumption (43).

We used the software R to search food additives and Stata v.16.1 for data analysis.

## 3. Results

**FIGURE 1:**
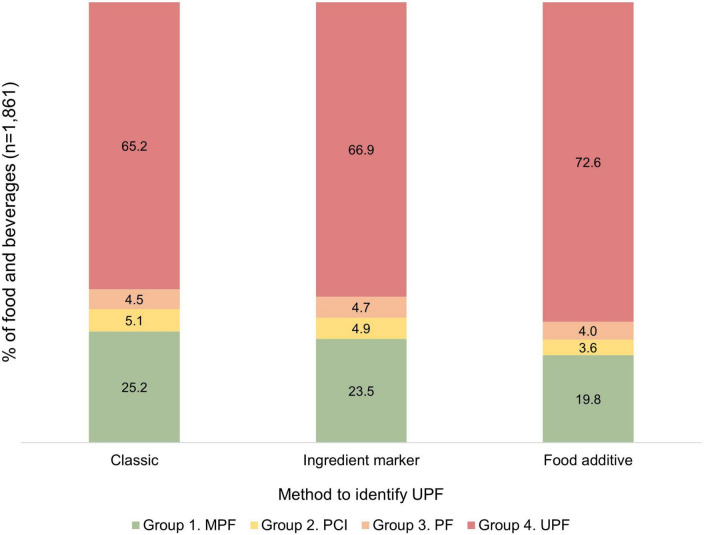
Proportion (%) of NOVA food groups using three methods to identify UPF in foods and beverages (*n* = 1,861). MPF, minimally processed foods; PCI, processed culinary ingredients; PF, processed foods; UPF, ultra-processed foods. In “classic method,” UPF was identified by using food description; in “ingredient marker method,” by searching for substances not commonly used in traditional recipes and names of functional classes of cosmetic additives in the lists of ingredients; and in “food additive method” by searching for UPF ingredient markers, names of functional classes and all individual names of cosmetic additives.

We identified 1,861 unique foods and beverages consumed by FECHIC children in 2016. The proportions of UPF varied with the different methods applied, especially when using food additives names. With the “classic method,” we classified 65.2% of foods as UPF, but this proportion increased when using more detailed ingredient information, reaching 66.9% with the “ingredient marker method” and 72.6% with the “food additive method” (Figure 1). From the former group of UPF, 30.7% had only cosmetic additives, being the most common emulsifiers (78.3%), thickeners (74.2%), flavorings (71.9%), and colors (60.3%) (data not shown).

Despite differences in the proportion of UPF, the agreement between the “classic” and “ingredient marker” methods, and the “ingredient marker” and “food additive” methods was almost perfect (k = 0.91 and k = 0.88, respectively), while the “classic” and “food additive” methods presented substantial agreement (k = 0.79) (Table 1). Sensitivity analyses conducted only including packaged foods produced similar agreement rates (Supplementary Tables 2–4).

**TABLE 1:** Agreement (%) and kappa coefficient between NOVA food groups obtained using three methods to identify UPF in foods and beverages (*n* = 1,861).

Method	Classic	Ingredient marker	Food additive
Classic	100; 1		
Ingredient marker	95.5; 0.91	100; 1	
Food additive	90.0; 0.79	94.3; 0.88	100; 1

In “classic method,” UPF was identified by using food description; in “ingredient marker method,” by searching for substances not commonly used in traditional recipes and names of functional classes of “cosmetic” additives in the lists of ingredients; and in “food additive method” by searching for UPF ingredient markers, names of functional classes and all individual names of cosmetic additives. In bold, the perfect agreement and kappa coefficient between a method and itself.

In Table 2 we explore the discrepancies among methods. Comparing the “classic” and “ingredient marker” methods, we observed that most of the differences were due to foods previously classified as MPF and then as UPF (8.1% of classic MPF; *n* = 38) and foods classified as PF and then as UPF (17.9% of classic PF; *n* = 15). Conversely, when using the “food additive” method we observed changes involving all NOVA groups: 22.6% of classic MPF (*n* = 106), 30.5% of classic PCI (*n* = 29), and 32.1% of classic PF (*n* = 27) were classified as UPF when we used all possible cosmetic food additives in the classification.

**TABLE 2:** Distribution (*n*; %) of NOVA food groups obtained in “classic method” according to “ingredient marker” and “food additive” methods in foods and beverages (*n* = 1,861).

Methods	Ingredient marker				Food additive			
Classic	Group 1. MPF	Group 2. PCI	Group 3. PF	Group 4. UPF	Group 1. MPF	Group 2. PCI	Group 3. PF	Group 4. UPF
Group 1. MPF (*n* = 469)	430 (91.7)	0 (0.0)	1 (0.2)	38 (8.1)	360 (77.2)	0 (0.0)	1 (0.2)	106 (22.6)
Group 2. PCI (*n* = 95)	0 (0.0)	90 (94.7)	1 (1.1)	4 (4.2)	0 (0.0)	65 (68.4)	1 (1.1)	29 (30.5)
Group 3. PF (*n* = 84)	0 (0.0)	0 (0.0)	69 (82.1)	15 (17.9)	0 (0.0)	0 (0.0)	57 (67.9)	27 (32.1)
Group 4. UPF (*n* = 1,213)	7 (0.6)	1 (0.1)	16 (1.3)	1.189 (98.0)	6 (0.5)	1 (0.1)	16 (1.3)	1.190 (98.1)

MPF, minimally processed foods; PCI, processed culinary ingredients; PF, processed foods; UPF, ultra-processed foods. In “classic method,” UPF was identified by using food description; in “ingredient marker method,” by searching for substances not commonly used in traditional recipes and names of functional classes of “cosmetic” additives in the lists of ingredients; and in “food additive method” by searching for UPF ingredient markers, names of functional classes and all individual names of “cosmetic” additives. In bold, the combination of same NOVA group in different methods.

Table 3 describes the proportion of UPF using different classification methods by food categories. In milk and plain yogurt, cheese, and cereals, flours, and pulses, we observed that more than half of the food products would change from non-UPF to UPF when using ingredients or additive markers. In snacks, fruits and vegetables preserves, and fats and oils, the proportion of UPF varied by about 20% depending on the UPF method used (Table 3). To provide an idea of the relative importance of each of these food categories in the study sample, we present the mean energy intake of FECHIC children by food category in Supplementary Table 5.

**TABLE 3:** Proportion (%) of UPF in food and beverages using three methods to identify them, according to food categories (*n* = 1,861).

Food categories	Classic	Ingredient marker	Food additive
Water, tea, and coffee (*n* = 46)	6.5	8.7	13.0
Sweetened beverages (*n* = 186)	98.4	99.5	99.5
Milk and plain yogurt (*n* = 53)	3.8	49.1	60.4
Milk-based drinks (*n* = 62)	100.0	100.0	100.0
Flavored or sweetened yogurt (*n* = 89)	100.0	100.0	100.0
Dairy desserts (*n* = 63)	98.4	96.8	96.8
Cheese (*n* = 36)	5.6	33.3	66.7
Cereals, flours, and pulses (*n* = 111)	7.2	6.3	62.2
Breakfast cereals, and granola bars (*n* = 73)	97.3	95.9	95.9
Fresh breads (*n* = 8)	0.0	0.0	0.0
Packaged breads (*n* = 35)	100.0	100.0	100.0
Crackers and cookies (*n* = 108)	100.0	98.1	98.1
Cakes and pies (*n* = 75)	100.0	100.0	100.0
Snacks (*n* = 51)	80.4	60.8	60.8
Confectionaries (*n* = 188)	100.0	98.4	98.4
Fast food (*n* = 16)	93.8	93.8	93.8
Soups, sauces, and salts (*n* = 85)	78.8	81.2	92.9
Meat, fish and eggs (*n* = 103)	1.0	1.0	1.0
Salted, smoked or canned meat or fish (*n* = 39)	59.0	61.5	61.5
Reconstituted meat or fish (*n* = 92)	98.9	100.0	100.0
Fruits and vegetables (*n* = 155)	0.6	0.6	0.6
Fruits and vegetable preserves (*n* = 53)	60.4	77.4	77.4
Baby food (*n* = 11)	54.5	54.5	54.5
Sweeteners (*n* = 36)	58.3	58.3	61.1
Fats and oils (*n* = 87)	31.0	32.2	47.1
Total (*n* = 1,861)	65.2	67.0	72.6

**FIGURE 2:**
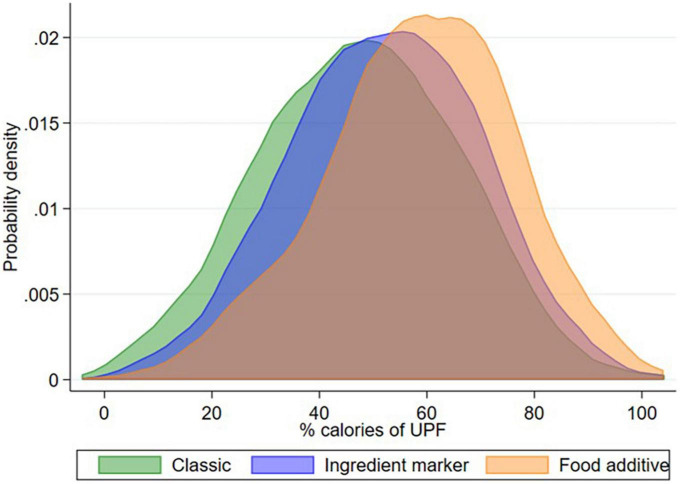
Probability density of caloric share of UPF in preschoolers’ diet using three methods to identify UPF. Food Environment Chilean Cohort (FECHIC) (*n* = 958). In “classic method,” UPF was identified by using food description; in “ingredient marker method,” by searching for substances not commonly used in traditional recipes and names of functional classes of “cosmetic” additives in the lists of ingredients; and in “food additive method” by searching for UPF ingredient markers, names of functional classes and all individual names of “cosmetic” additives.

We also observed differences in the caloric share of UPF in children’s diets when using the three methods to identify UPF. The caloric share of UPF was 47.4, 52.0, and 58.4% when using the “classic,” “ingredient marker,” and “food additive” methods, respectively (Table 4). Figure 2 shows a right displacement of the caloric share of UPF when we used either ingredients or additives to identify UPF. The density curves obtained for the “classic” and “ingredient marker” methods were similar in their symmetry and kurtosis; for the “food additive method,” the density curve was sharper and more left-tailed than the others. Despite the differences in the caloric share observed between the three methods, overall AA-ICC was 0.74 (95% CI0.56–0.84) and CA-ICC was 0.81 (95% CI 0.80–0.83), indicating moderate to good and good consistency. Measures of agreement were higher for the comparison between “classic” and “ingredient marker” method (AA-ICC: 0.80 [95% CI 0.71–0.86]; CA-ICC: 0.83 [95% CI 0.81–0.85]) than between classic and additive-bases method (AA-ICC: 0.62 [95% CI 0.20–0.80]; CA-ICC: 0.73 [95% CI 0.70–0.76]).

**TABLE 4:** Caloric share (%; 95% confidence interval) of NOVA food groups in preschoolers’ diet using three methods to identify UPF.

Method	Group 1. MPF	Group 2. PCI	Group 3. PF	Group 4. UPF
Classic	34.2 (33.2–35.2)	7.9 (7.5–8.2)	10.5 (9.9–11.1)	47.4 (46.2–48.6)
Ingredient marker	29.4 (28.5–30.3)	7.7 (7.3–8.0)	10.9 (10.2–11.5)	52.0 (50.9–53.2)
Food additive	23.7 (22.8–24.5)	7.2 (6.9–7.6)	10.7 (10.1–11.3)	58.4 (57.3–59.5)

Food Environment Chilean Cohort (FECHIC) (n = 958). MPF, minimally processed foods; PCI, processed culinary ingredients; PF, processed foods; UPF, ultra-processed foods. In “classic method,” UPF was identified by using food description; in “ingredient marker method,” by searching for substances not commonly used in traditional recipes and names of functional classes of “cosmetic” additives in the lists of ingredients; and in “food additive method” by searching for UPF ingredient markers, names of functional classes and all individual names of “cosmetic” additives.

When comparing quintiles of the dietary share of UPF by classification method, we found a higher proportion of agreement in the fifth and first quintiles for both comparisons: 71.7 and 65.1% for “classic” and “ingredient marker,” and 60.7% and 54.7% for “classic” and “food additive” method (Table 5). We observed substantial (weighted kappa = 0.65) and moderate agreement (weighted kappa = 0.51) between “classic” and “ingredient marker” and “classic” and “food additive” methods, respectively.

**TABLE 5:** Distribution (*n*; %) of quintiles of the caloric share of ultra-processed foods (% of total calories) in preschoolers’ diet according to different methods to identify UPF.

Classic method	Ingredient marker method*				
	Q1 (<37.1%)	Q2 (37.1–47.5%)	Q3 (47.6–57.1%)	Q4 (57.2–67.4%)	Q5 (>67.5%)
Q1 (<30.7%)	125 (65.1)	38 (19.8)	15 (7.8)	8 (4.2)	6 (3.1)
Q2 (30.7–42.4%)	59 (30.7)	87 (45.3)	22 (11.5)	16 (8.3)	8 (4.2)
Q3 (42.5–52.1%)	6 (3.1)	58 (30.4)	94 (49.2)	18 (9.4)	15 (7.9)
Q4 (52.2–64.0%)	1 (0.5)	8 (4.2)	53 (27.6)	105 (54.7)	25 (13)
Q5 (> 64.1%)	1 (0.5)	1 (0.5)	7 (3.7)	45 (23.6)	137 (71.7)
**Classic method**	Food additive method**				
	**Q1 (<43.8%)**	**Q2** **(43.9–54.4%)**	**Q3** **(54.5–64.0%)**	**Q4** **(64.1–73.4%)**	**Q5 (>73.5%)**
Q1 (<30.7%)	105 (54.7)	48 (25)	23 (11.9)	9 (4.7)	7 (3.7)
Q2 (30.7–42.4%)	68 (35.4)	56 (29.2)	33 (17.2)	25 (13)	10 (5.2)
Q3 (42.5–52.1%)	14 (7.3)	69 (36.1)	50 (26.2)	39 (20.4)	19 (10)
Q4 (52.2–64.0%)	4 (2.1)	17 (8.9)	77 (40.1)	55 (28.6)	39 (20.3)
Q5 (>64.1%)	1 (0.5)	2 (1.1)	8 (4.2)	64 (33.5)	116 (60.7)

Food Environment Chilean Cohort (FECHIC) (n = 958).

*Agreement = 86.1%; Weighted kappa = 0.65.

**Agreement = 80.3%; Weighted kappa = 0.51.

In “classic method,” UPF was identified by using food description; in “ingredient marker method,” by searching for substances not commonly used in traditional recipes and names of functional classes of “cosmetic” additives in the lists of ingredients; and in “food additive method” by searching for UPF ingredient markers, names of functional classes and all individual names of “cosmetic” additives.

## 4. Discussion

Our results indicate that using ingredient information for applying NOVA food classification system increased the proportion of food and beverages classified as UPF. However, despite the observed differences, we found almost perfect agreement between the “classic” and “ingredient marker” methods, and substantial agreement between “classic” and “food additive” methods in classifying food products. When applied to dietary data of Chilean preschoolers, we observed that the mean caloric share of UPF increased by 5% when we included information from ingredient markers and 11% when we included food additives compared to estimates based on food description (i.e., classic method). However, we found good consistency and absolute agreement for the caloric share of UPF among the three methods. The agreements for UPF quintiles were substantial and moderate for “classic” vs. “ingredient marker” and “classic” vs. “food additive” methods, respectively.

To our knowledge, this is the first study that reports how different UPF assessment methods shift the proportion of UPF in food products and dietary share. Previous studies have compared the consistency of the NOVA classification system between different raters. In a study conducted in the United States, two Ph.D. level researchers used the food item description to apply NOVA and two other food processing classifications on the 100 foods most consumed by children who participated in the National Health and Nutrition Examination Survey 2013–2014 (44). The authors found a lower agreement with NOVA than with the other classifications. In France, in an online survey, more than 100 specialists in food and nutrition classified two lists of foods into NOVA groups, and the consistency among evaluators both for a list of generic foods and for marketed foods with lists of ingredients was low (Fleiss’ kappa coefficient around 0.3) (24). Conversely, in our study, we performed inter-rater reliability using food description to apply NOVA (our classic method) in 5% of all products of SER-24 (n = 306) and found almost perfect agreement. This finding suggests that trained raters might have a better classification consistency.

In our study, most differences between the “classic” and “ingredient marker” methods were due to foods that were classified as MPF or PF in the classic method and then as UPF when we searched for ingredient markers. Exploring the lists of ingredients, we found that fruit preserves with and without added sugars were classified as UPF in the “ingredient marker method” because they had concentrated juice, coloring, or thickener. Many fluids and powdered milk previously classified as MPF included emulsifiers. Other discrepancies were found in cheeses with coloring or gelatin. We also found a small number of foods classified as UPF in the “classic method” and then as PF (about 1% of products). Some condensed milk, for instance, was classified as UPF when we applied the “classic method,” but as PF with the use of the “list of ingredients method” because they were only made of milk and sugar. Among snacks, reported differences were because some potato chips were only made of potato, oil, salt, and antioxidants, and classified as PF by the “ingredient marker” method. Including food additive names in the search resulted in about a quarter of foods from the other “classic” NOVA categories (MPF, PCI, and PF) to the UPF group. For cereals, most of the disagreement was due to the food additive riboflavin found in pasta. Riboflavin is a vitamin that can also be used as coloring (37). For fats and oils, the difference was explained by the presence of polydimethylsiloxane, an additive that could be an emulsifier, antifoaming, or anticaking agent (37). In salts, we found silicon dioxide, an additive that could be antifoaming, anticaking, or a carrier agent (37).

Applying the NOVA classification based on the list of ingredients could be a more objective procedure to identify a UPF. When the NOVA developers proposed a list of markers of UPF, they were attempting to solve an issue in the differentiation of processed and ultra-processed foods in some categories in which it is possible to find both types of processing as bakery products (3). However, in our study, extensively searching for possible cosmetic additives in the list of ingredients resulted in the identification of products that do not represent the concept of UPF. Our results showed that a third of the packaged products (30.6%) were classified as UPF only by the presence of a cosmetic additive in the “food additive” method (i.e., these products did not present a non-additive marker of UPF), including some milk, cheese, cereals, and oils. These products are usually classified as minimally processed, culinary ingredients, or processed foods because they contain whole foods and ingredients that we usually use in our kitchens.

Our findings suggest that the extensive use of food additives seems to result in an excessive proportion of products classified as UPF. This scenario was probably due to the large variety of functional classes of additives indicated as cosmetics by NOVA’s proposing authors (12 of 27 classes of Codex Alimentarius) and because many food additives could have different uses. Additionally, the extensive list of approved food additives makes their use for food classification difficult since they are not always declared with the exact name and code available in Codex. Finally, our experience indicates that using food additives could not be done routinely for researchers and policymakers interested in applying the NOVA food classification system. Searching for all food additives was time-consuming and code intensive. Then, to identify ultra-processed foods and inform consumers (i.e., using a warning label) (45, 46) or for other types of regulatory policies, it is necessary a clearer definition of UPF, with fewer but more consistent markers, that could potentially vary by food category. To specify these markers, it is also relevant consider that “cosmetic additives” is not a definition stated in the Codex Alimentarius, what could be a barrier to their use in regulations.

On the other hand the three methods applied were highly consistent in the identification of UPF in food categories such as soft drinks, breads, cookies, cakes and pies, milk-based drinks, and confectionaries, which represent a substantial part of UPF consumption in different countries (25, 33, 47). Overall, we identified 69.3% of UPF searching only for non-additive marker and about 99.9% using non-additive markers plus sweeteners, colors, and flavorings (data not shown). However, even using a few functional classes of additives to identify UPF should be considered with caution, because some vitamins and minerals used for fortification can also be considered cosmetic additives. This is the case of riboflavin, calcium carbonate and carotenes—all classified as colors according to the Codex Alimentarius (37). Flavorings also deserve to be dealt with caution. Despite being commonly used in foods (48), they are not a functional class of additives described by the Codex Alimentarius (37). Most classic UPF without a non-additive marker (i.e., requiring the presence of a food additive to be identified) were soft drinks, milk-based beverages, and confectionaries (data not shown). These products are commonly cited as examples of UPF and could be classified as UPF when the list of ingredients are not available (3).

The differences in the identification of UPF affected the estimated caloric share of UPF in the FECHIC preschoolers. The extension of disagreements is at least partly explained by the importance of specific food categories in the energy intake of our participants. Most disagreements in the frequency of UPF were found in milk and plain yogurt and cereals, flours, and pulses, and these categories also contributed to most of the energy intake of our sample (Supplementary Table 5). Besides the differences observed between the three methods, we found moderate to good agreement between them by analyzing children’s diets. Particularly, we found good consistency of agreement, which indicates that the values were systematically correlated (42). Because there is no recommendation on tolerable or adequate consumption levels of UPF, authors usually compare quartiles or quintiles of the dietary share of UPF in the population’s diets to study associations between UPF consumption and health outcomes (1, 49). Using quintiles of dietary share of UPF of Chilean preschoolers, we found better agreements in the first and fifth quintiles. Thus, our findings suggest that the use of lists of ingredients and food additives for applying NOVA food classification could impact greater the description of the consumption of UPF than epidemiological studies in which associations are reported comparing the fifth and the first quintile. However, further analyses would be relevant to assess the exact impact of misclassification, particularly in populations where the consumption of dairy products or cereals are important UPF sources.

This study has some limitations. We could not consider the specific use of the food additive for each product as this information was not always available in the package. Instead, we decided to consider all functional classes an additive could assume. Then, an additive was defined as cosmetic if listed by the Codex Alimentarius in any of the twelve classes indicated by Monteiro et al. (3). Further, different products available in the food supply but not consumed by our participants were not included in our study, and our results may not be generalizable to high-income children or adults. On the other hand, our study has several strengths. We used detailed dietary data, which included the brand and flavor of industrialized foods and beverages. This information helped us apply the “classic method” to identify UPF and allowed us to match food items with a database containing ingredient information. We linked most foods and beverages with updated package information collected in supermarket chains with the largest sales volumes in Santiago in the same year of dietary food collection. We also searched for more than 350 food additives described by the Codex Alimentarius and included multiple synonyms described in the Chilean regulation or found in the packaged products database.

In conclusion, searching for all possible markers of UPF in the list of ingredients increased the proportion of UPF in food products, particularly in some food categories; and those differences affected the overall caloric share of UPF in the Chilean preschoolers’ diet. The current definition of UPF considers terms that are not stated in international and widely used food regulatory documents such as the Codex Alimentarius (e.g., “cosmetic additive”), nor have clear definitions such as substances with no or unusual use in home cooking. These limitations make the classification of UPF more prone to be disputed when they are an essential part of regulatory or legal processes. Taking into consideration a clearer range of other attributes of UPF besides their ingredients can contribute to a more unbiased definition of UPF for food policies.

## Data availability statement

The data used in this article are available upon reasonable request directed to CC, ccorvalan@inta.uchile.cl.

## Ethics statement

This study was reviewed and approved by the Ethics Committees of Institute of Nutrition and Food Technology (INTA) and the School of Public Health, University of Chile. Written informed consent to participate in this study was provided by the participants’ legal guardian/next of kin.

## Author contributions

CZR: conceptualization, methodology, formal analysis, writing—original draft, and writing—review and editing. AD: methodology and writing—review and editing. MG: writing—review and editing. NR and XD-T: writing—review and editing. MR: funding acquisition. CC: conceptualization, methodology, writing—review and editing, and funding acquisition. All authors contributed to the article and approved the submitted version.
