# Supplementary material for: Impact of the use of food ingredients and additives on the estimation of ultra-processed foods and beverages
Source: Front Nutr. 2023 Jan 10;9:1046463. doi: 10.3389/fnut.2022.1046463 (PMC9872514; doi:10.3389/fnut.2022.1046463)
Supplement: Supplementary file 1 [file Table_1.DOCX]

S1. Descriptions of food categories used in the study.

| Food category | Description |
| --- | --- |
| Water, tea, and coffee | Includes tap water, noncarbonated and carbonated bottled water, tea bags, and powdered or instant coffee. |
| Sweetened beverages | Includes sodas, fruit-based beverages (nectars and others), powder/concentrated drinks, and sports and energy drinks with or without sugar. |
| Plain milk and yogurt | Includes plain whole, semi-skimmed, and skimmed liquid and powdered milk, and plain yogurt. |
| Dairy drinks | Includes dairy drinks, flavored milk, and fermented milk with and without sugar. |
| Flavored yogurts | Includes flavored yogurt with or without sugar. |
| Dairy desserts | Includes ice-creams, milkshakes, dulce de leche, mousse, flan, and powder for dairy desserts. |
| Cheese | All types of cheese. |
| Cereals, flours, and pulses | Includes pasta, rice, corn, oat, flour, beans, lentils, and peas. |
| Breakfast cereals and granola bars | Includes sweetened breakfast cereals and granola bars. |
| Breads | Includes packaged or fresh bread. |
| Crackers and cookies | Includes salty crackers and sweet cookies. |
| Cakes and pies | Includes cakes, brownies, alfajores, donuts, sweet pies, and sweet bread. |
| Snacks | Includes salty and sweet snacks made from grain, starchy vegetables, or tuber. Includes snacks of nuts and seeds and popcorn. |
| Confectionaries | Includes bonbons, bubble gums, candy pops, chewy candies, chocolate-covered dried fruits, chocolates, gummies, jellies, caramel bars, caramel pops, marshmallows, and flavoring powders for milk. |
| Fast foods | Includes burgers, pizza, chicken, fries, and empanadas. |
| Soups, sauces, and salts | Includes instant soups, vinegar, ketchup, mustard, mayo, other sauces, bouillon cubes, and salt. |
| Meat and eggs | Includes fresh and frozen chicken, pork, beef, fish, seafood, and eggs. |
| Processed meats | Includes canned seafood and fish, chicken, pork, beef, or turkey hamburgers, nuggets, sausages, ham, and other processed meats. |
| Fresh and frozen fruits and vegetables | Includes fresh and frozen fruits and vegetables (including natural juices), herbs, algae, and mushrooms. |
| Fruits and vegetable preserves | Fruits preserved in syrup or fruit juice, jam, marmalade, dried fruit, and vegetable preserved in vinegar and/or oil and salt. |
| Baby food | Includes baby food products, pureed, with cereals, fruits, vegetables, and meats. Includes infant formula and supplements. |
| Sweeteners | Sugar and non-caloric sweeteners, artificial or natural. |
| Fats and oils | Includes oils and fats from plants, seeds, or animal sources, including butter, milk cream, and margarine. |
