# Supplementary material for: Impact of the use of food ingredients and additives on the estimation of ultra-processed foods and beverages
Source: Front Nutr. 2023 Jan 10;9:1046463. doi: 10.3389/fnut.2022.1046463 (PMC9872514; doi:10.3389/fnut.2022.1046463)
Supplement: Supplementary file 2 [file Table_2.DOCX]

Table S2. Proportion (%) of NOVA food groups obtained using three methods to identify UPF in packaged foods and beverages (n=1,449).

| Method | Group 1. MPF | Group 2. PCI | Group 3. PF | Group 4. UPF |
| --- | --- | --- | --- | --- |
| Classic | 13.9 | 6.4 | 4.2 | 75.5 |
| Ingredient marker | 11.7 | 6.1 | 4.4 | 77.8 |
| Food additive | 6.9 | 4.4 | 3.6 | 85.1 |

Notes: MPF: minimally processed foods, PCI: processed culinary ingredients, PF: processed foods, UPF: ultra-processed foods. In ‘classic method’, UPF was identified by using food description; in ‘ingredient marker method’, by searching for substances not commonly used in traditional recipes and names of functional classes of cosmetic additives in the lists of ingredients; and in ‘food additive method’ by searching for UPF ingredient markers, names of functional classes and all individual names of cosmetic additives.
