# Supplementary material for: Impact of the use of food ingredients and additives on the estimation of ultra-processed foods and beverages
Source: Front Nutr. 2023 Jan 10;9:1046463. doi: 10.3389/fnut.2022.1046463 (PMC9872514; doi:10.3389/fnut.2022.1046463)
Supplement: Supplementary file 3 [file Table_3.DOCX]

Table S3. Agreement (%) and kappa coefficient between NOVA food groups obtained using three methods to identify UPF in packaged foods and beverages (n=1,449).

| Method  Method | Classic | Ingredient marker | Food additive |
| --- | --- | --- | --- |
| Classic | 100; 1 |  |  |
| Ingredient marker | 94.3; 0.85 | 100; 1 |  |
| Food additive | 87.1; 0.62 | 92.7; 0.78 | 100; 1 |

Notes: In ‘classic method’, UPF was identified by using food description; in ‘ingredient marker method’, by searching for substances not commonly used in traditional recipes and names of functional classes of ‘cosmetic’ additives in the lists of ingredients; and in ‘food additive method’ by searching for UPF ingredient markers, names of functional classes and all individual names of cosmetic additives.
