# Supplementary material for: Impact of the use of food ingredients and additives on the estimation of ultra-processed foods and beverages
Source: Front Nutr. 2023 Jan 10;9:1046463. doi: 10.3389/fnut.2022.1046463 (PMC9872514; doi:10.3389/fnut.2022.1046463)
Supplement: Supplementary file 4 [file Table_4.DOCX]

Table S4. Proportion (%) of UPF in packaged food and beverages using three methods to identify them, according to food categories (n=1,449).

| **Food categories** | **Classic** | **Ingredient marker** | **Food additive** |
| --- | --- | --- | --- |
| Water, tea, and coffee (n=40) | 7.5 | 10.0 | 15.0 |
| Sweetened beverages (n=185) | 98.9 | 100.0 | 100.0 |
| Milk and plain yogurt (n=52) | 3.8 | 50.0 | 61.5 |
| Milk-based drinks (n=51) | 100.0 | 100.0 | 100.0 |
| Flavored or sweetened yogurt (n=88) | 100.0 | 100.0 | 100.0 |
| Dairy desserts (n=57) | 98.2 | 96.5 | 96.5 |
| Cheese (n=28) | 7.1 | 42.9 | 85.7 |
| Cereals, flours, and pulses (n=101) | 7.9 | 6.9 | 68.3 |
| Breakfast cereals, and granola bars (n=72) | 97.2 | 95.8 | 95.8 |
| Fresh breads (n=0) | - | - | - |
| Packaged breads (n=29) | 100.0 | 100.0 | 100.0 |
| Crackers and cookies (n=108) | 100.0 | 98.1 | 98.1 |
| Cakes and pies (n=31) | 100.0 | 100.0 | 100.0 |
| Snacks (n=40) | 92.5 | 67.5 | 67.5 |
| Confectionaries (n=184) | 100.0 | 98.4 | 98.4 |
| Fast food (n=2) | 100.0 | 100.0 | 100.0 |
| Soups, sauces, and salts (n=83) | 78.3 | 80.7 | 92.8 |
| Meat, fish and eggs (n=2) | 50.0 | 50.0 | 50.0 |
| Salted, smoked or canned meat or fish (n=29) | 48.3 | 51.7 | 51.7 |
| Reconstituted meat or fish (n=78) | 98.7 | 100.0 | 100.0 |
| Fruits and vegetables (n=10) | 300.0 | 390.0 | 390.0 |
| Fruits and vegetable preserves (n=47) | 12.8 | 12.8 | 12.8 |
| Baby food (n=11) | 181.8 | 181.8 | 190.9 |
| Sweeteners (n=34) | 79.4 | 82.4 | 120.6 |
| Fats and oils (n=87) | 0.0 | 0.0 | 0.0 |
| Total (n=1,449) | 75.5 | 77.8 | 85.1 |
