# Supplementary material for: Impact of the use of food ingredients and additives on the estimation of ultra-processed foods and beverages
Source: Front Nutr. 2023 Jan 10;9:1046463. doi: 10.3389/fnut.2022.1046463 (PMC9872514; doi:10.3389/fnut.2022.1046463)
Supplement: Supplementary file 5 [file Table_5.DOCX]

Table S5. Distribution of mean energy intake in preschoolers' diet by food categories. Food Environment Chilean Cohort (FECHIC) (n=958).

| **Food categories** | **kcal** | **95% IC** | | **Caloric share**  **(% kcal)** |
| --- | --- | --- | --- | --- |
| Water, tea, and coffee | 0.05 | 0.01 | 0.08 | 0.0 |
| Sweetened beverages | 68.50 | 63.38 | 73.62 | 5.8 |
| Milk and plain yogurt | 122.77 | 114.03 | 131.51 | 10.4 |
| Milk-based drinks | 87.44 | 79.79 | 95.09 | 7.4 |
| Flavored yogurts | 72.23 | 66.49 | 77.98 | 6.1 |
| Dairy desserts | 12.99 | 10.03 | 15.95 | 1.1 |
| Cheese | 15.41 | 13.01 | 17.82 | 1.3 |
| Cereals, flours, and pulses | 136.03 | 127.80 | 144.26 | 11.5 |
| Breakfast cereals, and granola bars | 37.67 | 33.70 | 41.63 | 3.2 |
| Fresh breads | 97.25 | 90.98 | 103.52 | 8.2 |
| Packaged breads | 15.76 | 12.31 | 19.20 | 1.3 |
| Crackers and cookies | 70.48 | 62.19 | 78.76 | 6.0 |
| Cakes and pies | 33.52 | 26.86 | 40.17 | 2.8 |
| Snacks | 23.58 | 17.93 | 29.23 | 2.0 |
| Confectionaries | 43.07 | 37.47 | 48.66 | 3.6 |
| Fast food | 9.70 | 6.19 | 13.21 | 0.8 |
| Soups, sauces, and salts | 7.27 | 5.75 | 8.78 | 0.6 |
| Meat, fish and eggs | 76.44 | 71.85 | 81.02 | 6.5 |
| Salted, smoked or canned meat or fish | 9.58 | 7.49 | 11.68 | 0.8 |
| Reconstituted meat or fish | 30.65 | 25.82 | 35.48 | 2.6 |
| Fruits and vegetables | 98.85 | 93.10 | 104.60 | 8.4 |
| Fruits and vegetable preserves | 9.43 | 7.64 | 11.22 | 0.8 |
| Baby food | 3.39 | 1.35 | 5.43 | 0.3 |
| Sweeteners | 13.17 | 11.15 | 15.19 | 1.1 |
| Fats and oils | 86.66 | 82.16 | 91.16 | 7.3 |
| Total | 1181.87 | 1158.00 | 1205.80 | 100.0 |
